# Prognostic Scores for Acute Kidney Injury in Critically Ill Patients

**DOI:** 10.3390/nursrep14040264

**Published:** 2024-11-20

**Authors:** Wisble Pereira Sousa, Marcia Cristina da Silva Magro, Alberto Augusto Martins Paiva, Ruth Silva Rodrigues Vasconcelos, Abraão Alves dos Reis, Wellington Luiz de Lima, Tayse Tâmara da Paixão Duarte

**Affiliations:** 1 Faculty of Health Sciences and Technology, University of Brasília/UnB, Brasília 72220-275, Brazil; wisblesousa@gmail.com (W.P.S.); marciamagro@unb.br (M.C.d.S.M.); ruth.silva@aluno.unb.br (R.S.R.V.); abraao.reis@aluno.unb.br (A.A.d.R.); 2 Postgraduate Program in Nursing, University of Brasília, Brasília 70910-900, Brazil; albertopaiva19@hotmail.com; 3 Health Department of the Federal District, Brasília 72215-270, Brazil; wellington.lima@saude.df.gov.br

**Keywords:** critical care, nursing, organ dysfunction scores, acute kidney injury, intensive care units, predictive value

## Abstract

Background: Numerous prognostic scores have been developed and used in intensive care; however, the applicability and effectiveness of these scores in critically ill patients with acute kidney injury may vary due to the characteristics of this population. Objective: To assess the predictive capacity of the Simplified Acute Physiology Score III (SAPS III), Sequential Sepsis-related Organ Failure Assessment (SOFA) and Nursing Activities Score (NAS) prognostic scoring systems for acute kidney injury in critically ill patients. Methods: Cohort, prospective and quantitative study with follow-up of 141 critical patients in intensive care. A questionnaire was used to collect information about the capacity of prognostic scoring systems to predict AKI. Mann–Whitney, Kruskal–Wallis and Bonferroni-corrected Mann–Whitney tests were used and the statistical significance was considered to be at two-sided *p* < 0.05. Results: It was revealed that 41.85% of patients developed acute kidney injury during their stay in the Intensive Care Unit and indicated greater severity assessed by the medians of prognostic scoring systems—SAPS III [55 (42–65 vs. 38 (32–52), *p* < 0.001], SOFA [3.3 (2.26–5.00) vs. 0.66 (0.06–2.29), *p* < 0.001] and NAS [90 (75–95) vs. 97 (91–103), *p* < 0.001]—when compared to patients without kidney damage. Conclusions: The SAPS III, SOFA and NAS prognostic scoring systems showed good predictive capacity for acute kidney injury in critically ill patients. This study was not registered.

## 1. Introduction

Acute Kidney Injury (AKI) represents a complex clinical condition characterized by the sudden decline in kidney function, resulting in the inability of the kidneys to excrete metabolic products and maintain the body’s fluid and electrolyte balance [1]. This syndrome is frequently observed in critically ill patients and is associated with a series of adverse complications that include increased morbimortality, prolonged hospitalization and increased healthcare costs [2].

There has been a high incidence of AKI in recent decades, which represents a significant public health concern as it impacts millions of patients worldwide annually and affects approximately 45.4% of patients admitted to Intensive Care Units (ICU) [3], reflecting factors such as the increasing complexity of patients admitted to ICUs, the increased survival of patients with critical illnesses and the advances in diagnostic and monitoring methods [4].

The severity of critically ill patients with AKI is a significant concern due to their negative and often adverse outcomes [5]. The presence of AKI in critically ill patients further aggravates their clinical condition, as it increases the risk of cardiovascular complications, fluid and electrolyte disorders and the need for invasive therapies [6,7].

Nevertheless, numerous prognostic scores have been developed and used in intensive care, covering a variety of clinical features and laboratory data [8]. However, the applicability and effectiveness of these scores in critically ill patients with AKI may vary due to the unique characteristics of this population such as the presence of multiple comorbidities, hemodynamic instability and exposure to intensive therapies [9].

The Simplified Acute Physiology Score 3 (SAPS 3), the Sequential Organ Failure Assessment (SOFA) Score and the Nursing Activities Score (NAS) have been used as prognostic evaluation systems to measure severity, incorporating different clinical, laboratorial and physiological variables to provide an estimate of illness severity, the risk of mortality and the nursing workload for critical patients [10].

In this context, this study aimed to assess the predictive capacity of the Simplified Acute Physiology Score III, Sequential Sepsis-related Organ Failure Assessment and Nursing Activities Score prognostic scoring systems for acute kidney injury in critically ill patients, providing valuable insights regarding risk stratification, clinical decision-making and optimization of treatment for these patients. By better understanding the usefulness and accuracy of these scores among patients with AKI, we hope to contribute to a more effective and personalized approach regarding the care of these individuals in the ICU environment.

## 2. Materials and Methods

### 2.1. Type of Study

Cohort, prospective study with a quantitative approach according to STROBE (Strengthening the Reporting of Observational Studies in Epidemiology) recommendations.

### 2.2. Place and Period of Data Collection

This study was carried out from December 2022 to June 2023 with patients admitted to a 19-bed adult clinical and surgical Intensive Care Unit (ICU) of a public teaching hospital in the Midwest region of Brazil.

### 2.3. Population, Selection Criteria and Sampling

All adult patients aged 18 years or over, who remained hospitalized in the Intensive Care Unit for a minimum of 48 h were included. Patients who previously underwent kidney transplant and/or showed creatinine clearance values of less than 30 mL/min/1.73 m^2^ were excluded (Glomerular filtration rate was estimated using baseline serum creatinine values and the Chronic Kidney Disease Epidemiology Collaboration—CKD-EPI equation) [11].

The estimated glomerular filtration rate (GFR) at baseline was calculated for each hospital admission with the CKD-EPI equation, which uses serum creatinine as the main marker for calculating GFR = 141 × min (SCR/k, 1)^α^ × max (SCR/k, 1)^−1.209^ × 0.993^Age^ × 1.018 [if female] × 1.159 [if black], where κ equals 0.7 for women and 0.9 for men, α corresponds to 0.329 for women and 0.411 for men, min indicates the minimum of CrS/κand 1 and max indicates the maximum of CrS/κf and 1 [11]. Creatinine levels were measured using Jaffé’s kinetic method with cap and without deproteinization.

**Figure 1:**
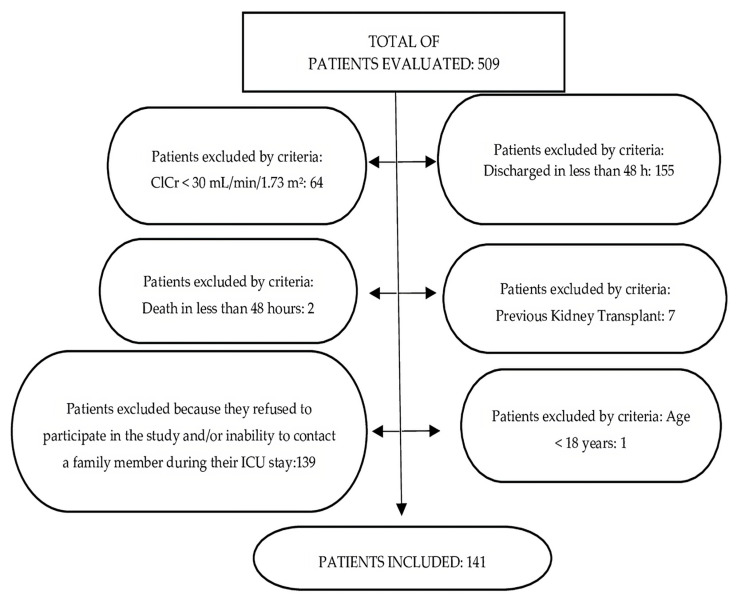
Flowchart of the sample size used in the research.

Follow-ups of 509 patients admitted to the ICU were conducted. After evaluating the inclusion/exclusion criteria, 141 patients were recruited. Sample losses were based on the following factors: less than 48 h of stay in the ICU, creatinine clearance values < 30 mL/min/1.73 m^2^, previous kidney transplant, death in less than 48 h of hospitalization, age < 18 years, refusal to participate in the research and/or inability to contact family members during ICU stay (Figure 1).

The sample size calculation considered 80% power, and the following formula was adopted [12]:

$$2\frac{{\left[z_{\alpha }2{\left(\bar{p}\bar{q}\right)}^{\frac{1}{2}}+z_{\beta }{\left(p1q1+p2q2\right)}^{\frac{1}{2}}\right]}^{2}(1+(n-1)\rho )}{n{\left(p1-p2\right)}^{2}}$$

where p1 corresponds to the proportion of individuals who had complete recovery from renal failure in the first level of the categorical variable; p2 refers to the proportion of individuals who had complete recovery from renal failure in the second level of the categorical variable; q1 = 1 − p1; q2 = 1 − p2; $\bar{p}$ = (p1 + p2)/2; $\bar{q}$ = 1 − $\bar{p}$; ρ refers to the intraclass correlation; n is number of measurements made on the same individual; z_α_ is the percentile of the normal distribution corresponding to the significance level; and z_β_ is the percentile of the normal distribution corresponding to the power of the test.

### 2.4. Research Instrument and Variables

Patient data were recorded using an instrument created by the researchers and included information focused on clinical and demographic characteristics such as sex (male and female), age, Body Mass Index (BMI), ethnicity (absent data, white, black, brown, indigenous and yellow), marital status (absent data, single, married, widower and divorced), comorbidities (diabetes, high blood pressure, cancer, heart diseases, chronic kidney disease and liver diseases), left ventricular ejection fraction, reason for admission to the ICU (clinical or surgical) and length of stay in the ICU.

The instrument items also aimed to determine whether the patient needed renal replacement therapy (hemodialysis) before and/or during their stay in the ICU or whether they needed a blood transfusion during their stay in the ICU. Other aspects were included: the need for mechanical ventilation and the time spent on it, medications taken during their ICU stay (vasoactive drugs, antibiotics and diuretics) and laboratory tests (creatinine, hematocrit and hemoglobin).

Furthermore, nursing workload was investigated using the Nursing Activities Score (NAS) [13], and patient severity was analyzed using the Simplified Acute Physiology Score III (SAPS III) [14] and the Sequential Sepsis-related Organ Failure Assessment (SOFA) prognostic scoring systems [15].

The NAS scoring system allows the assessment of the nursing team’s workload for each patient, indicating the work demand over a 24 h period, evaluating patients on ventilatory support and assessing their cardiovascular, renal, neurological and metabolic functions; basic activities; and interventions carried out. Each item scores from 1.2 to 32 points, which are added together at the end and can reach a maximum score of 176.8 points [13]. NAS data were recorded once a day by nurses working in this ICU.

As for SAPS III, it predicts the mortality of patients admitted to the ICU by assessing 20 criteria (age; comorbidity; length of hospital stay prior to ICU admission; in-hospital ward prior to ICU admission; use of vasoactive drugs prior to admission; if hospitalization was planned; reasons for admission; site of surgery; if there was acute infection on admission; Glasgow coma scale; body temperature; heart rate; systolic pressure; total bilirubin; creatinine; leukocytes, pH, platelets and oxygenation) [14].

The SOFA scoring system, in turn, assesses organ dysfunction and can infer the morbidity of heterogeneous groups of individuals admitted to intensive care by assessing six organ systems (respiratory, cardiovascular, hepatic, coagulation, renal and neurological) [15].

The renal function assessment was based on the serum creatinine criterion established in the KDIGO (Kidney Disease: Improving Global Outcomes) guidelines due to the lack of a systematic and accurate record of urinary volume, which classifies the severity of AKI into the following stages: Stage 1—serum creatinine value greater than or equal to 0.3 mg/dL; Stage 2—a 2.0- to 2.9-fold increase in serum creatinine level compared to baseline level; Stage 3—a 3-fold increase in the serum creatinine level compared to baseline level, creatinine value ≥ 4 mg/dL or start of renal replacement therapy [16]. The baseline creatinine level applied was based on the following strategies: (1) lowest value obtained in the first 7 days of ICU stay; (2) creatinine value at hospital admission; (3) creatinine value at ICU admission (4); lowest creatinine value in the period from 7 to 365 days prior to hospital admission [17,18].

The parameters for laboratory tests followed those recommended by the ICU where the study was conducted: plasma creatinine (sCr) (0.5–0.9 mg/dL), hemoglobin (Hb) (11.7 to 15.7 g/dL) and hematocrit (Ht) (35.0 to 47.0%).

### 2.5. Data Collection and Analysis

Data collection was conducted in 03 (three) stages:

Stage I: Weekly evaluation of clinical and laboratory records found in physical and electronic medical files to recruit patients according to the following criteria: 18 years of age or older, at least 48 h of stay in the ICU and creatinine clearance values greater than 30 mL/min/1.73 m^2^.

Stage II: Voluntarily obtaining free and informed consent by consulting the patient’s relative in charge. The impossibility of a face-to-face approach due to the family member’s absence led to a telephone consultation, with up to five attempts on different days. If this contact attempt was unsuccessful, the patient was excluded from the study. These measures were adopted in accordance with the recommendations of the Ethics and Research Committee of the University of Brasília, following resolution No. 510/2016.

Stage III: Patient follow-up from the date of admission to the ICU to their discharge from the ICU, discharge from the hospital, death or transfer to another hospital unit. Follow-up occurred for a maximum period of 20 days.

Data were typed into Microsoft^®^ Windows Excel^®^ 2019 spreadsheets, version 16.0 (Microsoft^®^, New Mexico, United States) and analyzed with the R programming environment (version 4.3.2). Descriptive measures such as mean, median, standard deviation, interquartile range, absolute frequency and percentages were used to describe the characteristics of the variables under study and provide summary information about the data collected. Chi-square and Fisher’s exact tests were used to verify statistically significant associations between the variables.

The evaluation of missing data to identify possible biases was carried out using the Mann–Whitney, Chi-Square and Fisher’s Exact tests. Sensitivity analyses were performed to explore the impact of missing or imputed values of (1) baseline sCr, (2) body weight and (3) daily sCr on our model. Additional sensitivity analyses were performed to evaluate how alternative definitions of baseline sCr affected our model. We considered the following alternative definitions: (1) lowest value of the first 7 days of ICU stay (2); hospital admission creatinine; (3) ICU admission creatinine; (4) lower creatinine value in the period from 7 to 365 days before hospital admission [17,18].

The Shapiro–Wilk test identified that the data presented were not parametric and, therefore, the Mann–Whitney, Kruskal–Wallis and Bonferroni-corrected Mann–Whitney tests were used to compare the medians of two independent samples, three or more independent samples and to make multiple comparisons, respectively, in situations where the data failed to meet the assumptions of normal distribution and homogeneity of variance.

Unadjusted models and adjusted models were constructed and, for the latter, the Backward Selection method was applied with a 5% permanence level. In the present study, all statistical analyses were performed using the R programming environment version 4.3.2 (R core team 2023, Auckland, New Zealand).

### 2.6. Ethical Aspects

In accordance with Resolutions No. 466/2012 and No. 510/2016, and following Report No. 3.327.399 approved on 15 May 2019, the Ethics and Research Committee of the University of Brasília approved this study. Informed consent was obtained from all subjects involved in the study.

## 3. Results

Of the 141 patients, almost half (41.85%) developed acute kidney injury (AKI) and the majority developed more severe AKI (KDIGO 2 and 3) (75%) during their stay in the Intensive Care Unit (ICU).

The median age of patients with AKI was lower—57 (48–66) years old—than that of those without AKI, 61 (50–70) years old, but they still required a longer median length of hospitalization [9 (5.3–14.0) days vs. 5 (3.0–8.0) days, <0.001]. The use of vasoactive drugs was significant in the group with AKI compared to the group without AKI [73% vs. 33%, *p* < 0.001], as well as the use of antibiotics [86% vs. 50%, *p* < 0.001], diuretics [36% vs. 17%, *p* = 0.0012] and invasive mechanical ventilation support [61% vs. 20%, *p* < 0.001] (Table 1).

The left ventricular ejection fraction (LVEF) of the left ventricle was significantly lower in the group with AKI when compared to the group without AKI [45 (30, 52) vs. 60 (48, 64), *p* = 0.002], as well as hemoglobin level [9.21 (8.50–11.03) g/dL vs. 12.03 (9.64–13.55) g/dL, *p* < 0.001] and hematocrit average value [28 (25–33)% vs. 36 (29–40)%, *p* < 0.001] (Table 1).

Patients with AKI showed greater severity when assessed by prognostic scores, with a median SAPS III value of [55 (42–65) vs. 38 (32–53), *p* < 0.001], a median SOFA value of [3.3 (2.26–5.00) vs. 0.66 (0.06–2.29), *p* < 0.001] and a median NAS value of [97 (91, 103) vs. 90 (75, 95), *p* < 0.001] when compared to those without AKI (Table 1).

Some variables expressed an increased risk of developing AKI when evaluated independently. The use of vasoactive drugs increased the risk of AKI by 2.63 times, while the use of antibiotics tripled the risk (RR = 3.04) of developing AKI. Greater patient severity expressed by the NAS scoring system showed a higher risk for AKI [1.02 (1.00–1.03), *p* = 0.012] (Table 2).

**Figure 2:**
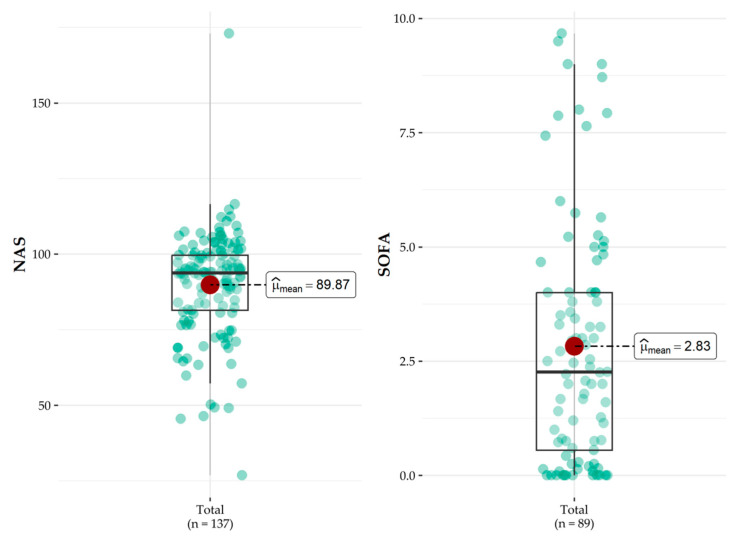
NAS and SOFA scores of critically ill patients in the Intensive Care Unit. Brasília, DF, Brazil, 2023.

Figure 2 shows the severity of patients expressed by the NAS and SOFA scoring systems. We can observe that the NAS distribution shows a mean score of 89.87, but several points are far from the center of distribution, concentrated between the median and the third quartile, thus expressing greater severity in most patients. Confirming the clinical severity profile of patients in this ICU, a mean SOFA value greater than 2 (2.83) was observed.

**Figure 3:**
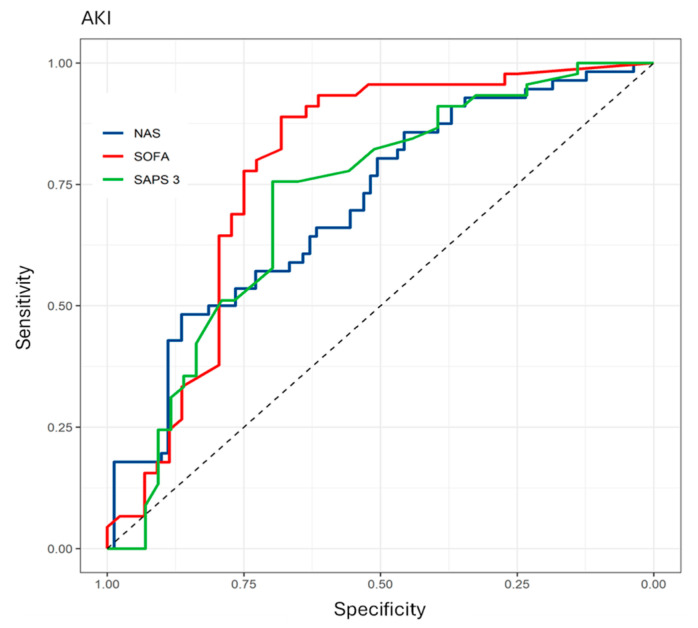
Risk prediction for the development of AKI by applying the NAS, SOFA and SAPS 3 scoring systems. Brasília, DF, Brazil, 2023.

The assessment of sensitivity and specificity showed good AKI prediction. Similar performance was observed in the Area Under the Curve (AUC) between the NAS, SOFA and SAPS III scores, with respective AUC values of 0.707 [95% CI 0.619–0.795, *p* < 0.001], 0.781 [95% CI 0.678–0.884, *p* < 0.001] and 0.717 [95% CI 0.607–0.828, *p* < 0.001] (Figure 3).

## 4. Discussion

The results obtained in this study reveal that the SOFA (AUC 0.781), SAPS III (0.717) and NAS (0.707) severity scores showed good capacity to predict AKI in critically ill patients, as evidenced by the AUC values above 0.7.

In this study, the SOFA and SAPS III scoring systems presented an acceptable capacity to predict the development of AKI, but the literature has presented controversial data on the predictive performance of mortality based on the use of these scoring systems [19]. In a study by Jung et al., conducted with 652 patients, the AUC value identified was 0.66 using the SOFA scoring system and 0.71 using the SAPS scoring system [19]. In a multicenter study conducted in China with 9079 patients, the AUC value identified was 0.686 using the SOFA scoring system and 0.767 using the SAPS scoring system [20]. To develop continuous and challenging mortality predictions in critically ill patients with AKI, scoring systems such as the SAPS III and the SOFA continue to be widely used in intensive care to guide clinical decisions and healthcare [21].

It is known that the discriminatory power of the NAS scoring system indicates a high demand for care by the nursing team, and the highest NAS value has been associated with the development of AKI in critically ill patients [22,23], as evidenced in this study.

It was also observed that patients with AKI had a significantly longer length of stay in the ICU compared to those without AKI. It is known that a longer hospital stay may be associated with the progression of AKI, greater clinical deterioration as well as metabolic changes resulting from kidney injury. It must be considered that among critically ill patients with AKI, the length of stay associated with a higher hospital mortality rate corresponds to a 30-day period [24].

Other findings of this study identified a lower left ventricular ejection fraction (LVEF) in patients with AKI. Persistent low cardiac output may result in decreased renal blood flow, predisposing them to AKI [25]. Increased need for mechanical ventilation is also associated with AKI, as shown in other scientific evidences [7,26], since this treatment is capable of causing renal function impairment due to the positive pressure that increases the intra-abdominal pressure and consequently reduces renal blood flow, in addition to inducing a systemic inflammatory response and increasing the production of reactive oxygen species and causing carbon dioxide retention, which may lead to respiratory acidosis and impact renal function [7].

The presence of lower hemoglobin and hematocrit values in patients with AKI when compared to those without renal dysfunction was also found, which can trigger vasoconstriction and reduced renal blood flow, thus increasing the risk of kidney injury [27]. It must also be considered that this occurrence may trigger the need for blood transfusion, a factor that was also proven to be associated with the development of AKI in this study and in other scientific evidences, given that volume overload may lead to renal overload, in addition to the fact that the transfused blood components may contain pro-inflammatory or pro-coagulant substances that trigger an inflammatory response in the body and increase the risk of clot formation within the renal vessels, thus causing renal obstruction and impairment [28].

It is known that the use of vasoactive drugs, antibiotics and diuretics in the ICU has a direct impact on renal function due to their nephrotoxicity, which was significantly confirmed in our results. The interaction of these drugs with the renal system is complex and multifaceted: vasoactive drugs may induce vasoconstriction of renal vessels, resulting in hypoperfusion and kidney ischemia [29]. Antibiotics, in turn, cause direct damage to the renal tubules [30] and, finally, diuretics may lead to dehydration and hypovolemia, resulting in reduced renal blood flow, which contributes to the occurrence of AKI [31]. These interactions highlight the importance of careful monitoring and management of these medications in patients at risk of developing AKI to mitigate their negative impact on renal function. Most patients (75%) with AKI were identified in stages 2 or 3 of the KDIGO classification—that is, with greater severity of renal impairment. Other studies [9,32] also perpetuate this idea that renal function in the intensive care environment is more impaired, which may be associated with the severity profile of critically ill patients, evidenced by the prolonged use of nephrotoxic drugs, need for mechanical ventilation, laboratory changes and need for blood transfusion, as observed in this study. All these aspects confirm the need for collaborative work by a multidisciplinary team and show the relevance of nurses’ professional practice.

Interprofessional collaboration has become an important component of a well-functioning health system [33]. Contemporary nursing practice is based on person-centered care, shared decision-making and multidisciplinary teamwork. Nurses, as the first point of contact with their patients and providing direct care, play a unique role in collaborating with members of the healthcare team [34].

This study faced some limitations. This is a single-center study; the urinary output criterion aimed at identifying and classifying AKI according to the KDIGO classification was not used due to data inaccuracy in the research unit; there was also a risk of measurement bias due to the fact that the data were collected in physical and electronic medical records, which may contain misreports.

## 5. Conclusions

The use of vasoactive drugs, antibiotics and diuretics; prolonged length of stay in the ICU; and prolonged mechanical ventilation were factors associated with the development of acute kidney injury (AKI) in critically ill patients. It was observed that the SAPS III, SOFA and NAS scoring systems had good predictive capacity for AKI.

In this context, we recommend the consideration that introducing AKI care bundles into routine clinical practice can effectively improve the outcomes of patients with or at risk of AKI. In addition, the implementation of prognostic indicators such as SAPS, SOFA and NAS in combination with advanced analytical models can make the assessment of the impacts of AKI in the critical patient setting more accurate. Also, we consider that machine learning and artificial intelligence have shown promise for identifying patterns and increasing the accuracy of clinical diagnoses of AKI.

## Figures and Tables

**Table 1:** Association between clinical, sociodemographic and laboratory variables and acute kidney injury in critically ill patients (n = 141). Brasília, DF, Brazil, 2023.

Variables	AKI *		*p*-Value
	No (N ^†^ = 82/58.15%)	Yes (N ^†^ = 59/41.85%)	
Age in years			0.249 ^1^
Mean (SD ^‡^)	59 (15)	56 (15)	
Median [IQR ^§^]	61 [50–70]	57 [48–66]	
Sex, n (%)			0.597 ^2^
Female	44 (54)	29 (49)	
Male	38 (46)	30 (51)	
Ethnicity, n (%)			0.910 ^3^
Yellow	1 (1.2)	2 (3.4)	
White	11 (13)	10 (17)	
Indigenous	1 (1.2)	0 (0)	
Black	55 (66.7)	39 (66.4)	
Absent data	14 (17)	8 (14)	
Marital status, n (%)			0.537 ^3^
Married	21 (26)	22 (37)	
Divorced	3 (3.7)	1 (1.7)	
Absent data	35 (43)	19 (32)	
Single	19 (23)	15 (25)	
Widower	4 (4.9)	2 (3.4)	
BMI ^‖^ (kg/m^2^)			0.878 ^1^
Mean (SD ^‡^)	25.5 (4.7)	25.9 (5.8)	
Median [IQR ^§^]	26.2 [23.3–28.4]	25.2 [22.2–29.0]	
Reason for admission to the ICU ^¶^, n (%)			0.965 ^2^
Surgical	40 (49)	29 (49)	
Clinical	42 (51)	30 (51)	
Length of stay (days)			<0.001 ^1^
Mean (SD ^‡^)	6.2 (4.3)	9.8 (5.1)	
Median [IQR ^§^]	5.0 [3.0, 8.0]	9.0 [5.3, 14.0]	
Renal Replacement Therapy (RRT)			
Before admission to the ICU ^¶^, n (%)	0 (0)	5 (8.5)	0.012 ^3^
During ICU stay ^¶^, n (%)	0 (0)	7 (12)	0.002 ^3^
Left ventricular ejection fraction			0.002 ^1^
Mean (SD ^‡^)	56 (13)	43 (16)	
Median [IQR ^§^]	60 [48–64]	45 [30–52]	
Comorbidities			
Diabetes, n (%)	36 (44)	17 (29)	0.068 ^2^
Hypertension, n (%)	57 (70)	33 (56)	0.098 ^2^
Cancer, n (%)	11 (13)	9 (15)	0.757 ^2^
Heart diseases, n (%)	46 (56)	27 (46)	0.226 ^2^
Chronic kidney disease, n (%)	7 (8.5)	9 (15)	0.215 ^2^
Liver diseases, n (%)	8 (9.8)	7 (12)	0.689 ^2^
Use of vasoactive drugs, n (%)	27 (33)	43 (73)	<0.001 ^2^
Catecholamines, n (%)	21 (26)	41 (69)	<0.001 ^2^
Vasodilators, n (%)	6 (7.3)	11 (19)	0.042 ^2^
Antidiuretic hormone, n (%)	1 (1.2)	12 (20)	<0.001 ^2^
Used antibiotics, n (%)	41 (50)	51 (86)	<0.001 ^2^
Polymyxin b, n (%)	1 (1.2)	7 (12)	0.010 ^3^
Beta-Lactams, n (%)	37 (45)	49 (83)	<0.001 ^2^
Glycopeptides, n (%)	6 (7.3)	22 (37)	<0.001 ^2^
Use of diuretics, n (%)	14 (17)	21 (36)	0.012 ^2^
Loop diuretics, n (%)	10 (12)	19 (32)	0.004 ^2^
Mechanical ventilation, n (%)	16 (20)	36 (61)	<0.001 ^2^
Time spent on mechanical ventilation (minutes)			0.003 ^1^
Mean (SD ^‡^)	2740 (4532)	7650 (7002)	
Median [IQR ^§^]	960 [645–2180]	4950 [1545–11,520]	
Blood transfusion, n (%)	8 (9.8)	20 (34)	<0.001 ^2^
Creatinine value at ICU admission ^¶^ (mg/dL^3^)			<0.001 ^1^
Mean (SD ^‡^)	0.97 (0.46)	1.87 (2.00)	
Median [IQR ^§^]	0.80 [0.70–1.08]	1.10 [0.88–1.93]	
>5	6/82 (7.3)	3/59 (5.1)	
Hemoglobin (g/dL)			<0.001 ^1^
Mean (SD ^‡^)	11.85 (2.48)	9.84 (1.92)	
Median [IQR ^§^]	12.03 [9.64–13.55]	9.21 [8.50–11.03]	
Hematocrit (%)			<0.001 ^1^
Mean (SD ^‡^)	35 (7)	29 (6)	
Median [IQR ^§^]	36 [29–40]	28 [25–33]	
SAPS 3 **			<0.001 ^1^
Mean (SD ^‡^)	42 (18)	54 (15)	
Median [IQR ^§^]	38 [32–53]	55 [42–65]	
SOFA ^††^			<0.001 ^1^
Mean (SD ^‡^)	1.76 (2.48)	3.88 (2.40)	
Median [IQR ^§^]	0.66 [0.06–2.29]	3.30 [2.26–5.00]	
NAS ^‡‡^			<0.001 ^1^
Mean (SD ^‡^)	86 (19)	96 (12)	
Median [IQR ^§^]	90 [75–95]	97 [91–103]	
KDIGO ^§§^, n (%)			<0.001 ^2^
1	0 (0)	15 (25)	
2	0 (0)	24 (41)	
3	0 (0)	20 (34)	
KDIGO ^§§^ categorized n/n (%)			<0.00 ^2^
0–1	82 (100)	15 (25)	
2–3	0 (0)	44 (75)	
Death, n (%)	5 (6.1)	5 (8.5)	0.742 ^3^

* AKI—Acute Kidney Injury; ^†^ N—Absolute Frequency; ^‡^ SD—Standard Deviation; ^§^ IQR—Interquartile Range; ^‖^ BMI—Body Mass Index; ^¶^ ICU—Intensive Care Unit; ** SAPS III—Simplified Acute Physiology Score III; ^††^ SOFA—Sequential Sepsis-related Organ Failure Assessment; ^‡‡^ NAS—Nursing Activities Score; ^§§^ KDIGO—Kidney Disease: Improving Global Outcomes; ^1^ Wilcoxon–Mann–Whitney rank-sum test; ^2^ Chi-square test of independence; ^3^ Fisher’s exact test.

**Table 2:** Multivariate analysis of clinical and laboratory variables and acute kidney injury in critically ill patients (n = 141). Brasília, DF, Brazil, 2023.

Characteristics	RR * (CI ^†^ 95%)	*p*-Value
Used vasoactive drugs	2.63 (1.48–4.91)	0.001
Catecholamines	2.82 (1.62–5.13)	<0.001
Antidiuretic hormone	2.44 (1.20–4.55)	0.008
Used antibiotics	3.04 (1.52–6.96)	0.004
Beta-lactam antibiotic	2.76 (1.45–5.80)	0.004
Glycopeptide antibiotic	2.38 (1.35–4.08)	0.002
Used diuretics	1.66 (0.95–2.83)	0.068
Loop diuretic	1.82 (1.02–3.13)	0.035
Mechanical ventilation	2.73 (1.60–4.78)	<0.001
Hemoglobin (g/dL)		
13–17	—	
<13	3.41 (1.23–9.43)	0.018
>17	0.00 (0.00 to Inf)	0.992
Hematocrit	0.93 (0.88–0.97)	<0.001
NAS ^‡^	1.02 (1.00–1.03)	0.012
KDIGO ^§^ categorized	5.93 (3.35–11.1)	<0.001
0–1		
2–3		

* RR—Relative Risk; ^†^ CI—Confidence Interval; ^‡^ NAS—Nursing Activities Score; ^§^ KDIGO—Kidney Disease: Improving Global Outcomes.

## Data Availability

All data obtained in this study are available in this article.

## Public Involvement Statement

No public involvement in any aspect of this research.

## Guidelines and Standards Statement

This manuscript was drafted against Strengthening the Reporting of Observational Studies in Epidemiology (STROBE).
